# The Role of Chaperone-Mediated Autophagy in Huntingtin Degradation

**DOI:** 10.1371/journal.pone.0046834

**Published:** 2012-10-11

**Authors:** Lin Qi, Xing-Ding Zhang, Jun-Chao Wu, Fang Lin, Jin Wang, Marian DiFiglia, Zheng-Hong Qin

**Affiliations:** 1 Department of Pharmacology and Laboratory of Aging and Nervous Diseases, Soochow University School of Pharmaceutical Science, Suzhou, China; 2 Laboratory of Cellular Neurobiology, Massachusetts General Hospital and Harvard Medical School, Charlestown, Massachusetts, United States of America; Emory University, United States of America

## Abstract

Huntington Disease (HD) is caused by an abnormal expansion of polyQ tract in the protein named huntingtin (Htt). HD pathology is featured by accumulation and aggregation of mutant Htt in striatal and cortical neurons. Aberrant Htt degradation is implicated in HD pathogenesis. The aim of this study was to investigate the regulatory role of chaperone-mediated autophagy (CMA) components, heat shock protein cognate 70 (Hsc70) and lysosome-associated protein 2A (LAMP-2A) in degradation of Htt fragment 1-552aa (Htt-552). A cell model of HD was produced by overexpression of Htt-552 with adenovirus. The involvement of CMA components in degradation of Htt-552 was determined with over-expression or silencing of Hsc70 and LAMP-2A. The results confirmed previous reports that both macroautophagy and CMA were involved in degradation of Htt-552. Changing the levels of CMA-related proteins affected the accumulation of Htt-552. The lysosomal binding and luminal transport of Htt-552 was demonstrated by incubation of Htt-552 with isolated lysosomes. Expansion of the polyQ tract in Htt-552 impaired its uptake and degradation by lysosomes. Mutation of putative KFERQ motif in wild-type Htt-552 interfered with interactions between Htt-552 and Hsc70. Endogenous Hsc70 and LAMP-2A interacted with exogenously expressed Htt-552. Modulating the levels of CMA related proteins degraded endogenous full-length Htt. These studies suggest that Hsc70 and LAMP-2A through CMA play a role in the clearance of Htt and suggest a novel strategy to target the degradation of mutant Htt.

## Introduction

Abnormal accumulation of misfolded and aggregated proteins in neurons is a hallmark of several neurodegenerative diseases. The mutant proteins of neurodegenerative diseases can cause dysfunction and death of neurons. Huntington's disease (HD) is an autosomal dominant neurodegenerative disease caused by an abnormal expansion of polyQ tract in the N-terminal huntingtin (Htt). HD is characterized by the accumulation and aggregation of mutant N-terminal Htt proteins in diseased neurons [1]. When the polyQ repeat expands above 35, disease will manifest, typically striking in the late 40 s [2]. The N-terminal fragment of Htt containing 552 amino acids (Htt-552) is a caspase 2/3 cleavage product and can be found in normal and HD patient brains. Htt-552 with an expanded polyQ repeats causes an aggressive HD-like disease in animal and cell models [3]. N-terminal Htt was reported to be degraded by macroautophagy and ubiquitin-proteasome pathways [4], [5]. Although the chaperone-mediated autophagy (CMA) has been proposed to be involved in degradation of Htt, the molecular process and regulatory mechanisms have not yet been fully characterized.

The major pathways of mutant protein clearance are the proteasomal and the lysosomal systems in cells. These protein degradation pathways are compromised with aging [6]–[8]. In several types of neurodegenerative diseases, aggregated mutant proteins impair function of the proteasome, leading to accumulation of these diseases causing proteins in affected neurons. In this circumstance, the inducible autophagic pathway is likely to be the primary mechanism involved in the degradation of misfolded or aggregated proteins [9]–[11]. Autophagy is a degradation pathway for long-lived cytoplasmic components using lysosomes [12]–[16]. Based on the way substrates are transported into lysosomes, autophagy is classified into three types: macroautophagy, microautophagy and CMA. Among them, CMA has received particular attention because of its selectivity in degrading substrates compared to other forms of autophagy. In contrast to macroautophagy, a highly specific subset of cytosolic proteins with a motif recognized by the heat-shock cognate protein of 70 kDa chaperone (Hsc70) is selectively degraded in lysosomes via CMA [17], [18]. Following binding of the chaperone-substrate complex to a lysosomal membrane receptor, lysosome-associated membrane protein 2A (LAMP-2A) [19], CMA substrate proteins are translocated into the lysosomal lumen for degradation by hydrolases [20].

The involvement of CMA in neurodegeneration has been illustrated in Parkinson's disease. A mutation of Parkin-1 affected degradation of α-synuclein through CMA, leading to accumulation and aggregation of α-synuclein and degeneration of nigral dopominergic neurons [21]. Furthermore, mutant Tau protein involved in Alzheimer's disease (AD) is also targeted to lysosomes via CMA [22]. The advantage of CMA for protein degradation is its relative substrate selectivity compared to macroautophagy.

Up to now, the degradation of Htt by macroautophagy has been investigated by several investigators [4], [17], [23], whereas the involvement of the chaperone-mediated autophagy has received less attention. The vacuole targeting (Cvt) pathway in yeast which is similar to the selective autophagy pathway may be involved in degradation of polyQ repeat Htt [24]. Some groups have reported the association of CMA and HD [25], [26]. It has been proposed that N-terminal Htt fragment may contain KFERQ-like motifs following phosphorylation and be degraded via CMA. However, the supporting evidence on interactions of Htt with CMA related proteins and uptake and degradation of Htt by lysosomes is still missing. In this paper, we used rat neuronal like cells and human cells that were infected with adenovirus containing human Htt-552 with 18 or 100 glutamines (Htt-552-18Q or Htt-552-100Q) and evaluated whether Htt-552 interacted with LAMP-2A and Hsc70 and was transported into lysosomes for degradation. The results suggest that Htt-552 can be recognized by Hsc70 through KFERQ-like pentapeptide motif and is transported into lysosomes via LAMP-2A.

## Materials and Methods

### Cell culture and transfection by plasmids

PC12 and Hela cell lines were purchased from Shanghai Institute of Biochemistry and Cell Biology (Shanghai, China), and were grown at 37°C in 5% CO_2_ in DMEM medium supplemented with 2 mmol/L L-glutamine and 10% fetal bovine serum (FBS). To remove the serum, cells were washed twice with phosphate buffered saline (PBS) before replacing the complete medium with serum-free medium. We observed no changes in cell viability up to 72 h after serum removal. To activate CMA, cells were transfected with pcDNA4-LAMP-2A and pcDNA4-Hsp70 (human, kindly provided by Dr. GH. Wang, Soochow University School of Pharmaceutical Science, China). pcDNA4-Hsc70-His was generated by PCR from the I.M.A.G.E. clone for Hsc70 (Clone ID: NM_153201) with: ATTGAATTCATGTCCAAGGGACCTGCAG (forward) and GCGCTCGAGTCAACCTCTTCAATGGTGG (reverse), and subsequently digested with BamH I and Xho II and cloned in to the BamH I and Xho I sites of pcDNA4/HisA (V862-20, Invitrogen Carlsbad, CA, USA). Transfection was performed with Lipofectamine 2000 according to the manufacturer's instructions (11668-019, Invitrogen, Carlsbad, CA, USA).

To inhibit CMA activity, knockdown of CMA critical components was performed with siRNAs. Small interference RNAs (siRNA) targeting the following mRNAs were: (1) LAMP-2A: 5-GACTGCAGTGCAGATGAAG-3; (2) Hsc70: 5-CAGCACGGAAAAGUCGAGA-3 and 5- UAAUUCUAAGUACAUUGAGACCAGC-3; (3) Hsp70: 5-UCCUGUGUUUGCAAUGUUGAA-3 of human and (1) LAMP-2A: 5-GACTGCAGTGCAGATGAAG-3; (2) Hsc70: 5′-GGAAUGUGCUCAUUUUUGATT-3′ and 5′-UCAAAAAUGAGCACAUUCCTT-3′; (3) Hsp70: 5′-GTCTGAACGTGCTGCGGATCATCAA-3′ of rat. The siRNA oligos used to target LAMP-2A, Hsc70 and Hsp70 genes were previously validated [26]–[31]. For transfection of siRNA, cells were plated in 9-cm dishes at 30% confluence, and siRNA duplexes (200 nM) were introduced into the cells using Lipofectamine 2000 (11668-019, Invitrogen, Carlsbad, CA, USA) according to the manufacturer's recommendations.

### Adenoviral vector construction and cell infection

N-terminal fragments of wt Htt (Htt-552-18Q) and mutant Htt (Htt-552-100Q) were cloned into pDC316 adenovirus shuttle plasmid. cDNAs were excised from their parental vectors using BamHI and XbaI and then ligated to BamHI/XbaI-digested pUC18, an intermediate vector. Then these cDNAs were ligated to BglII/SalI-digested pDC316. Two independent adenovirus shuttle plasmids, pDC316-Htt-552-18Q stop and pDC316-Htt-552-100Q stop were obtained.

Ad-Htt-18Q-552 stop and Ad-Htt-100Q-552 stop, were obtained through co-transfecting T293A cells with the backbone plasmid pBHG10 and the shuttle plasmids: pDC316-Htt-552-18Q stop and pDC316-Htt-552-100Q stop. Cytopathic effects happened at the 7th day. The cells were collected at the 10th day to obtain the first generation adenovirus. Then the first generation of adenoviruses was proliferated in 293T cells. The viruses are named Ad- Htt-552-18Qaa and Ad-Htt-552-100Qaa, respectively. The fourth generation adenovirus was used in later experiments. The titers were 5×10^9^ and 7×10^9^, respectively. In addition, Ad-null-GFP adenoviral vector was generated and proliferated following the same protocol. PC12 and Hela cells were incubated in 1640 medium with 5% FBS containing adenoviral vectors. The virus-containing medium was removed 5 h later and replaced with fresh medium with 10% FBS.

### Immunofluorescence

For immunofluorescence microscopic examination, cells were plated onto 12-mm polylysine-coated coverslips and cultured for 24 h, cells were treated with siRNA or drugs. Cells were washed in PBS, fixed with 4% paraformaldehyde in PBS at 4°C for 10 min, and then washed again with PBS. The cells were permeabilized with 0.25% Triton X-100, and were then blocked with 10% normal goat serum (NGS) for 15 min. Primary antibodies, diluted in PBS with 1% bovine serum albumin were added to the cells and incubated for overnight at 4°C. The coverslips were washed three times before incubation with fluorescence-conjugated secondary antibodies using the same procedure as for the primary antibodies. The coverslips were mounted on slides with mounting medium (F4680, Sigma-Aldrich, Saint Louis, MO, USA) and were examined with a laser scanning confocal microscope. (C1S1, Nikon, Tokyo, Japan). The following primary antibodies were used: rabbit polyclonal anti-LAMP-2A antibody (ab18528, Abcam, Cambridge, MA, USA); goat polyclonal antibody against cathepsin D (sc-6488, Santa Cruz, Santa Cruz, CA, USA), rabbit polyclonal anti-Hsc70 antibody (ab51052, Abcam, Cambridge, MA, USA) and mouse monoclonal antibody against Htt (MAB2166, Chemicon (Millipore), Billerica, MA, USA).

### Immunoprecipitation

For immunoprecipitation studies, cells in dishes were transfected using adenovirus Htt-552 and Lipofectamine 2000 with plasmids of LAMP-2A and Hsc70 for 6 h and then maintained in complete media for 48 h and 72 h. Lysates were centriffuged at 16,000 g at 4°C for 15 min, and the supernatant was collected in immunoprecipitation buffer (50 mM Tris, 274 mM NaCl, 5 mM KCl, 5 mM EDTA, 1% Triton X-100, 1 mM PMSF and a protease and phosphatase inhibitor cocktail), Supernatants were precleared with protein G and subsequently incubated with anti-Htt antibody, anti-LAMP-2A antibody or anti-Hsc70 antibody overnight, and then 20 µL protein G were added and incubated for 2 h at 4°C. Protein G beads were washed three times in immunoprecipitation buffer, resuspended in sample buffer with 5% beta-mercaptoethanol and heated at 95°C for 5 min. Then the samples were run on SDS-PAGE and blotted to nitrocellulose for standard Western blot analysis.

### Western blot analysis

Western blot analysis was performed as described previously [32]. Cells were harvested and rinsed twice with ice-cooled PBS and homogenized in buffer containing 10 mmol/L Tris-HCl (pH 7.4), 150 mmol/L NaCl, 1% Triton X-100, 1% sodium deoxycholate, 0.1% SDS, 5 mol/L edetic acid, 1 mmol/L PMSF, 0.28 U/L aprotinin, 50 mg/L leupeptin, 1 mmol/L benzamidine, 7 mg/L pepstain A. Protein concentrations were determined using the BCA kit (Pierce, USA). Thirty micrograms of proteins from each sample were subjected to electrophoresis on 10–12% SDS-PAGE gel using a constant current. Proteins were transferred to nitrocellulose membranes and incubated with the Tris-buffered saline containing 0.2% Tween-20 (TBST) and 3% non-fat dry milk for 3 h in the presence of one of the following antibodies: mouse monoclonal antibody against Htt (MAB2166, Chemicon (Millipore), Billerica, MA, USA); mouse monoclonal antibody against β-actin (A5441, Chemicon (Millipore), Billerica, MA, USA), rabbit polyclonal antibodies against Beclin-1 (Sc-11427, Santa Cruz) and LAMP-2A (ab18528, Abcam, Cambridge, MA, USA), rabbit polyclonal antibodies against Hsc70 (ab51052, Abcam, Cambridge, MA, USA) and Hsp70 (ab47455, Abcam, Cambridge, MA, USA), rabbit polyclonal antibody against Htt (Ab1, kindly provided by Dr. Marian DiFiglia, Massachusetts General Hospital, USA). Membranes were washed and incubated with horseradish peroxidase-conjugated secondary antibody in TBST containing 3% non-fat dry milk for 1 h. Immunoreactivity was detected with enhanced chemoluminescent autoradiography (ECL kit, RPN2232, Amersham, Piscataway, NJ, USA) according to the manufacturer's instructions. The levels of protein expression were quantitatively analyzed with Sigma Scan Pro 5. The results were normalized to the loading control β-actin.

### Isolation of lysosomes

Subcellular fractionation was performed with a self-forming Percoll gradient. All steps were done at 4°C. Mouse liver was minced and washed in PBS, liver tissue was suspended in homogenization buffer (0.25 M sucrose, 2 mM EDTA, and 10 mM Hepes [pH 7.4]) and homogenized with a tight-fitting glass homogenizer. The homogenate was centrifuged (800 g, 10 min) to remove unbroken cells and the nuclei. The supernatant was centrifuged (6800 g, 10 min) to remove the large organelles including heavy mitochondria. The supernatant was centrifuged (25000 g, 10 min) to obtain the light organelles including lysosomes. The precipitation layered over 10 ml of a 27% Percoll (17-0891-01, Pharmacia Inc, Piscataway, NJ, USA) solution in homogenization buffer, under layered with 0.5 ml of a 2.5 M sucrose solution. Centrifugation was done in a rotor (Optima L90K, Beckman, Brea, CA, USA) for 1.5 h at 35000 g. The layer of crude lysosomes of about 1.5 ml was collected at the bottom and then was centrifuged (100000 g, 60 min) to remove the other light organelles including light mitochondria at the bottom of the tube. The lysosomal solution was washed with PBS (18000 g, 25 min) to remove the sucrose at the bottom of the tube. Following isolation, the purity of lysosomes was detected by Hsp60, β-actin, LAMP-2A and Hsc70. Lysosomal integrity was verified by measuring the activity of ß-hexosaminidase, a lysosomal enzyme, in the incubation medium. Preparations with more than 10% broken lysosomes after isolation or more than 20% at the end of the incubation were discarded [33]. Lysosomal solution was divided equally to 4 portions. Each portion was incubated with the cell lysates containing none, null, Htt-552-18Q and Htt-552-100Q for 3 h.

### Recombinant plasmids and site-directed mutagenesis

The recombinant plasmid pcDNA3.1-Htt-552, containing the pcDNA3.1 (+) mammalian expression vector (V790-20, Invitrogen, Carlsbad, CA, USA) and cDNA encoding the human Htt-552 protein, was constructed using standard cloning methods and subsequently used as a template for mutagenesis. Mutations at sites 100D→R/103N→A and 248N→A/249E→K were constructed by the site-directed mutagenesis using Phusion DNA polymerase (M0535L, New England Biolabs, Ipswich, MA, USA), with following primers: TGTCAGACAATGAGCCACACGGCGTTTCTTGGTAGC (forward), GCTACC AA GAAACGCCGTGTGGCTCATTGTCTGACA (reverse) for 100D→R/103N→A; TAACAAAACCTTAATTTTAGCGTCATTTGCAAAATT (forward), AATTTTG CA AATGACGCTAAAATTAAGGTTTTGTTA (reverse) for 248N→A/249E→K. The products were treated with Dpn1 polymerase (ER1702, Fermentas, Glen Burnie, MA, USA) and were recovered by transformation into competent bacteria for amplification. Each was confirmed by nucleotide sequence analysis. Transfection of 293A cells with expression plasmids was performed using Lipofectamine 2000 (11668-019, Invitrogen, Carlsbad, CA, USA).

### Uptake and degradation of Htt-552 by lysosomes

Uptake of Htt-552 by isolated lysosomes was analyzed as described previously [20]. Briefly, freshly isolated lysosomes from mice liver were incubated with Htt-552 in MOPS buffer at 37°C for 5 and 10 min. Where indicated, lysosomes were pre-incubated with a cocktail of protease inhibitors for 10 min at 0°C. Lysosomes were collected by centrifugation, washed with PBS buffer, and subjected to SDS-PAGE and immunoblotting for Htt-552. Uptake was calculated from densitometric analysis by subtracting the amount of Htt-552 associated with lysosomes in the presence (protein bound to the lysosomal membranes and taken by lysosomes) and absence (protein bound to the lysosomal membranes) of protease inhibitors. To separate lysosomal membranes and matrix, lysosomes were disrupted by hypotonic shock where indicated. Briefly, collect the isolated lysosomes by centrifugation (25,000 g for 10 min), resuspend the pellet fraction in a hypotonic buffer (0.025 M sucrose), and after 30-min incubation on ice, spin the samples at 150,000 g for 30 min to recover the membrane fraction in the pellet and the lysosomal matrix in the supernatant fraction [34].

### FACScan Flow Cytometric Analysis

Cell viablility analysis was performed as described previously [35], [36]. For flow cytometric analysis, Hela cells transfected with empty vector, Ad- Htt-552-18Qaa and Ad-Htt-552-100Qaa for 120 h were trypsinized, washed in PBS, and resuspended in ice-cold 80% ethanol. Briefly, 2.5×10^5^ fixed cells were incubated in 250 µL propidium iodide solution (500 mg/mL propidium iodide in 3.8 mol/L sodium citrate at pH 7.0) and 250 µL RNase A (10 mg/mL prepared in 10 mmol/L Tris–HCl at pH 7.5) for 30 min at 37°C in the dark. The stained cells were filtered through the cell strainer caps of Falcon polystyrene round-bottomed tubes. DNA content was analyzed on a Becton Dickinson FACScan (Becton Dickinson, San Jose, CA, USA). The population of sub-G1 was determined using Cell Fit software (Becton Dickinson, San Jose, CA, USA). Data were collected from at least 20,000 cells.

### Statistical analysis

Statistical analysis was carried out by one-way analysis of variance (ANOVA) followed by Dunnett t-test. Differences were considered significant when *p*<0.05.

## Results

### CMA is involved in degradation of Htt-552

**Figure 1:**
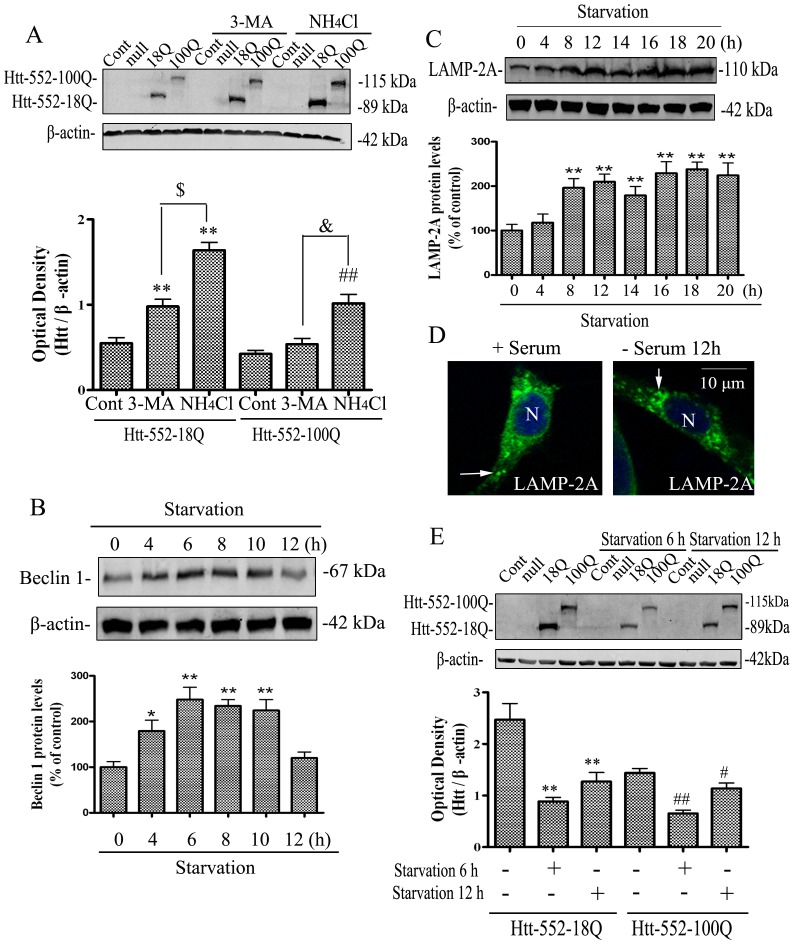
CMA is involved in degradation of Htt-552. (A) The effects of 3-MA and NH_4_Cl on the accumulation of Htt-552. Cells were transfected with Htt-552 for 48 h and then were treated with NH_4_Cl (15 mM) or 3-methyl adenine (3-MA, 10 mM) for 4 h. Cells were harvested and the effects of autophagy inhibitors on the accumulation of Htt-552 were determined with Western blot analysis. Values are the mean±SE of three independent experiments. **P<0.01 (compared with Htt-552-18Q without treatment); ^##^P<0.01 (compared with Htt-552-100Q without treatment); ^$^: p<0.05 (Htt-552-18Q+3-MA vs Htt-552-18Q+NH_4_Cl; &: p<0.05 ((Htt-552-100Q+3-MA vs Htt-552-100Q+NH4Cl); (B) The activations of macroautophagy induced by starvation as indicated by induction of Beclin 1. Starvation of PC12 cells were induced by removal of serum in the culture medium for indicated time. Values are the mean±SE of three independent experiments (*P<0.05; **P<0.01 vs 0 h). (C) The activations of CMA induced by starvation as indicated by induction of LAMP-2A. Values are the mean±SE of three independent experiments (**P<0.01 vs 0 h). (D) The relocation of CMA-active lysosomes toward the nuclear regions in PC12 cells. Cells were maintained in the presence or absence of serum. After 12 h, cells were fixed and subjected to immunofluorescence for LAMP-2A. Representative cells for each condition. N: nucleus. Thin arrows point to lysosomes. The scale bar represents 10 µm. (E) Effects of starvation for 6 or 12 h on the degradation of Htt-552 in PC12 cells. The effects of these starvations on protein levels of Htt-552 protein were determined with Western blot analysis. Values are the mean±SE of three independent experiments. **P<0.01 (compared with Htt-552-18Q without treatment); ^##^P<0.01, ^#^P<0.05 (compared with Htt-552-100Q without treatment).

The estimated rate of infection of adenovirus Htt-552 in PC12 or HeLa cells was over 95% (**Figure S1 A**). The expression of Htt-552-18Q and Htt-552-100Q in PC12 and HeLa cells was verified with Western blot analysis with anti-Htt antibodies 2166 and Ab1. Similar levels of wt and mutant Htt-552 were expressed in cells and the expression did not cause apparent cellular toxicity 48 h after infection (**Figure S2 A and B**). However, cell toxicity and apoptosis by mutant Htt was seen 120 h after infection and was exacerbated when CMA was compromised (**Figure S2 C and D**). To determine the involvement of lysosomes in degradation of Htt-552, PC12 cells were treated with ammonium chloride (NH_4_Cl, 15 mM), which inhibits lysosomal proteolysis. The results showed that ammonium chloride significantly inhibited the degradation of Htt-552. It was noted that the increase in accumulation of Htt-552-18Q was more robust than that of Htt-552-100Q (**Figure 1A**) in the presence of NH_4_Cl. To determine the contribution of macroautophagy to lysosomal degradation of Htt-552, PC12 cells were treated with 3-methyladenine (3-MA, 10 mM), an inhibitor of macroautophagy. The results demonstrated that 3-MA also reduced the degradation of Htt-552, but the effect was not as effective as ammonium chloride. It thus appeared that lysosomal degradation of exogenous Htt-552 could involve other autophagic mechanisms in addition to macroautophagy.

Starvation is a classical method to activate autophagy, including macroautophagy and chaperone-mediated autophagy (CMA). Serum removal markedly enhanced levels of Beclin 1, which is a macroautophagy regulatory protein, and LAMP-2A, which is a receptor protein of CMA (**Figure 1B and 1C**). The time-course of elevation of Beclin 1 was faster than that of LAMP-2A. When the levels of Beclin 1 returned to about basal levels, LAMP-2A reached its peak induction. The distance of the lysosomal vesicles to the nucleus is a useful indicator of activation of CMA [37]. An average of 4 different visual fields per group and 15 cells per visual field were evaluated. After starvation for 12 h, the distance of CMA-active LAMP-2A labeled lysosomes to the nucleus was reduced, indicating increased activity of CMA (**Figure 1D**).

Next, we examined the degradation of Htt-552 expressed in PC12 cells, at 6 h and 12 h after serum removal, the times at which there is maximal activation of macroautophagy and CMA, respectively. Removing serum for either 6 h or 12 h markedly reduced accumulation of Htt-552. However, Htt-552 levels appeared higher at 12 h than at 6 h (**Figure 1E**). These results suggested that wt and mutant Htt-552 could be degraded by both macroautophagy and CMA, but macroautophagy may contribute more.

### Association of Htt-552 and exogenously and endogenously expressed LAMP-2A and Hsc70

**Figure 2:**
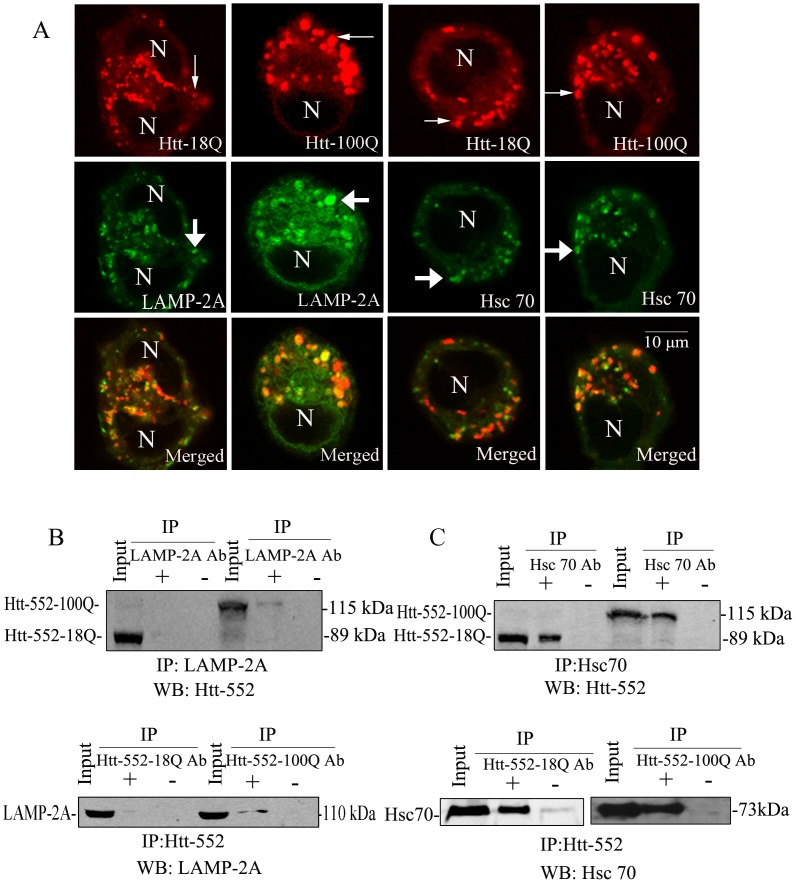
Association of Htt-552 and exogenous CMA component proteins. (A) Exogenous expressed LAMP-2A or Hsc70 co-localized with exogenous Htt-552-18Q and Htt-552-100Q in HeLa cells. Cells were transfected with Htt-552 for 48 h and fixed with methanol, blocked, and processed for double immunofluorescence with antibodies against LAMP-2A (green), Hsc70 (green) and Htt-552 (red). Merged images of both channels are shown at the bottom. N: nucleus. Thin arrows point to Htt-552 immunoreactivity. Thick arrows point to lysosomes. The scale bar = 10 µm. (B) Co-immunoprecipitation of exogenous expressed LAMP-2A with exogenous Htt-552. Upper panel: PC12 cell were transfected with PCDNA4-LAMP-2A for 72 h. Cell lysates were subjected to immunoprecipitation with anti-Htt antibody or no primary antibody and Western blot analysis was performed with anti-LAMP-2A antibody. Lower panel: PC12 cell were transfected with Htt-552 for 48 h. Cell lysates were subjected to immunoprecipitation with anti-LAMP-2A antibody or no primary antibody and Western blot analysis was performed with anti-Htt antibody. (C) Co-immunoprecipitation of exogenous expressed Hsc70 with Htt-552 from PC12 cells. Upper panel: PC12 cell were transfected with PCDNA4-Hsc70 for 72 h. Cell lysates were subjected to immunoprecipitation with anti-Hsc70 antibody or no primary antibody and Western blot analysis was performed with anti-Htt antibody. Lower panels:PC12 cell were transfected with Htt-552 for 48 h. Cell lysates were subjected to immunoprecipitation with anti-Htt antibody or no primary antibody and Western blot analysis was performed with anti-Hsc70 antibody. Ab: Antibody.

To characterize the association of exogenous Htt-552 clearance and CMA, the present study analyzed the co-localization of exogenously and endogenously expressed CMA component proteins LAMP-2A and Hsc70 with Htt-552 in HeLa cells. The results showed that the co-localization of immunoreactivities of exogenously and endogenously expressed LAMP-2A and Hsc70, two essential components of CMA, and Htt-552 was observed in cells expressing exogenous Htt-552 (**Figure 2A** **; Figure S3 A**). To verify the association of Htt-552 with LAMP-2A and Hsc70, co-immunoprecipitation of LAMP-2A or Hsc70 with Htt-552 was performed. In the cells expressing similar levels of wt and mutant Htt-552, exogenous and endogenous LAMP-2A or Hsc70 was co-immunoprecipitated with exogenous Htt-552 from PC12 cells (**Figure 2** **B and C; Figure S3 B and C**). There was a fair amount of Htt-552-100Q precipitated by LAMP-2A antibodies, however, there was little Htt-552-18Q precipitated by LAMP-2A (**Figure 2B** **and Figure S3 C**). These results confirm that Htt-552 proteins, especially Htt-552 with expanded polyQ tract can interact with proteins involved in CMA.

### Effects of LAMP-2A and Hsc70 on the accumulation of Htt-552

**Figure 3:**
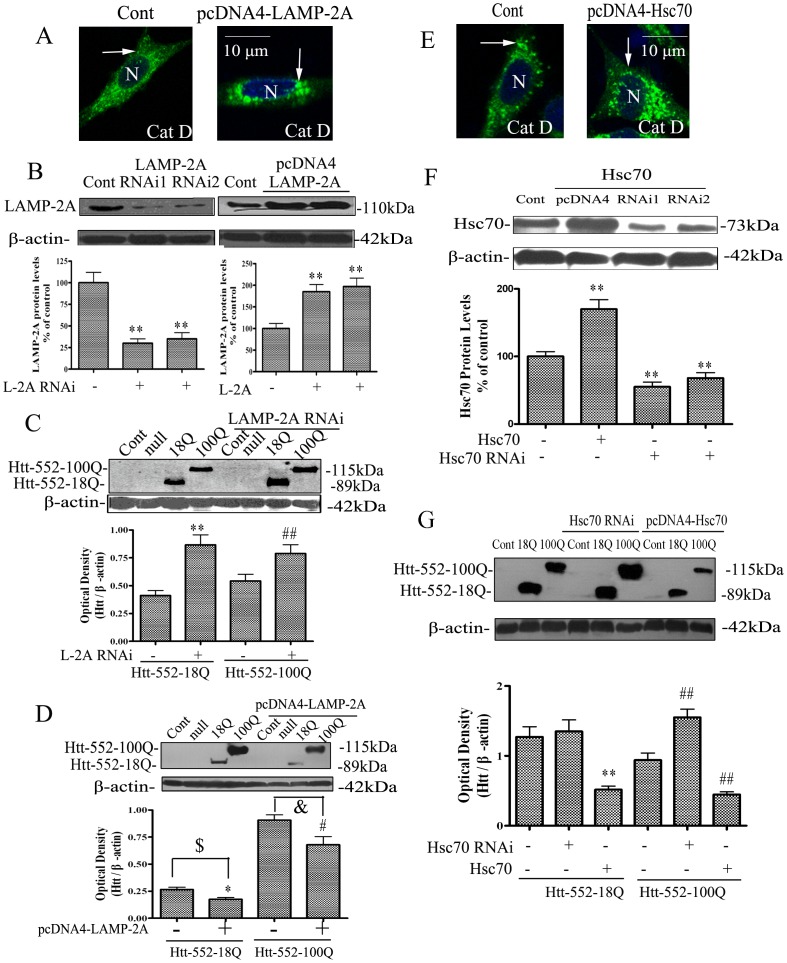
Change in CMA related proteins affects the accumulation of Htt-552. (A) Redistribution of CMA-active lysosomes induced by overexpression of LAMP-2A. PC12 cells were transfected with PCDNA4-LAMP-2A for72 h. Lysosomes were labeled with immunofluorescence of Cathepsin D (green) and the increased perineuclear localization of CMA-active lysosomes was observed. N: nucleus. Thin arrows point to lysosomes. The scale bar = 10 µm. (B) The efficiency of knockdown and overexpression of LAPM-2A. PC12 cells were transiently cotransfected with LAMP-2A plasmid or LAMP-2A siRNA for 48 h. Lysates were subjected to Western blot analysis with anti–human LAMP-2A. Values are the mean±SE of three independent experiments. **P<0.01 (compared with control); ^##^P<0.01 (compared with Htt-552-100Q with control). (C) and (D) Effects of knockdown and overexpression of LAMP-2A on Htt-552 levels. PC12 cells were transiently transfected with LAMP-2A plasmid, vector control or LAMP-2A siRNA for 72 h and Htt-552 for 48 h. Lysates were subjected to Western blot analysis with anti-Htt 2166, anti–human LAMP-2A antibodies. Values are the mean±SE of three independent experiments. *P<0.05 (compared with Htt-552-18Q without treatment); ^#^P<0.05 (compared with Htt-552-100Q without treatment). (E) Redistribution of CMA-active lysosomes induced by overexpression of Hsc70. PC12 cells were transfected with Hsc70 plasmid for 48 h. Lysosomes were labeled with immunofluorescence for Cathepsin D (green) in cultured PC12 cells and the re-location of CMA-active lysosomes toward the perinuclear regions was observed. N: nucleus. Thin arrows point to lysosomes. The scale bar represents 10 µm. (F) The efficiency of knockdown and everexpressiom of Hsc70. PC12 cells were transiently transfected with Hsc70 plasmid and Hsc70 siRNA for 48 h. Lysates were subjected to Western blot analysis with anti–Hsc70 antibody. Values are the mean±SE of three independent experiments (**P<0.01 vs control). (G) Effects of knockdown and overexpression of Hsc70 on Htt-552 levels. PC12 cells were transiently transfected with human Hsc70 plasmid, vector control or Hsc70 siRNA for 72 h and Htt-552 for 48 h. Lysates were subjected to Western blot analysis with anti-Htt 2166, anti–Hsc70 antibodies. Values are the mean±SE of three independent experiments. **P<0.01 (compared with Htt-552-18Q without treatment); ^##^P<0.01 (compared with Htt-552-100Q without treatment).

**Figure 4:**
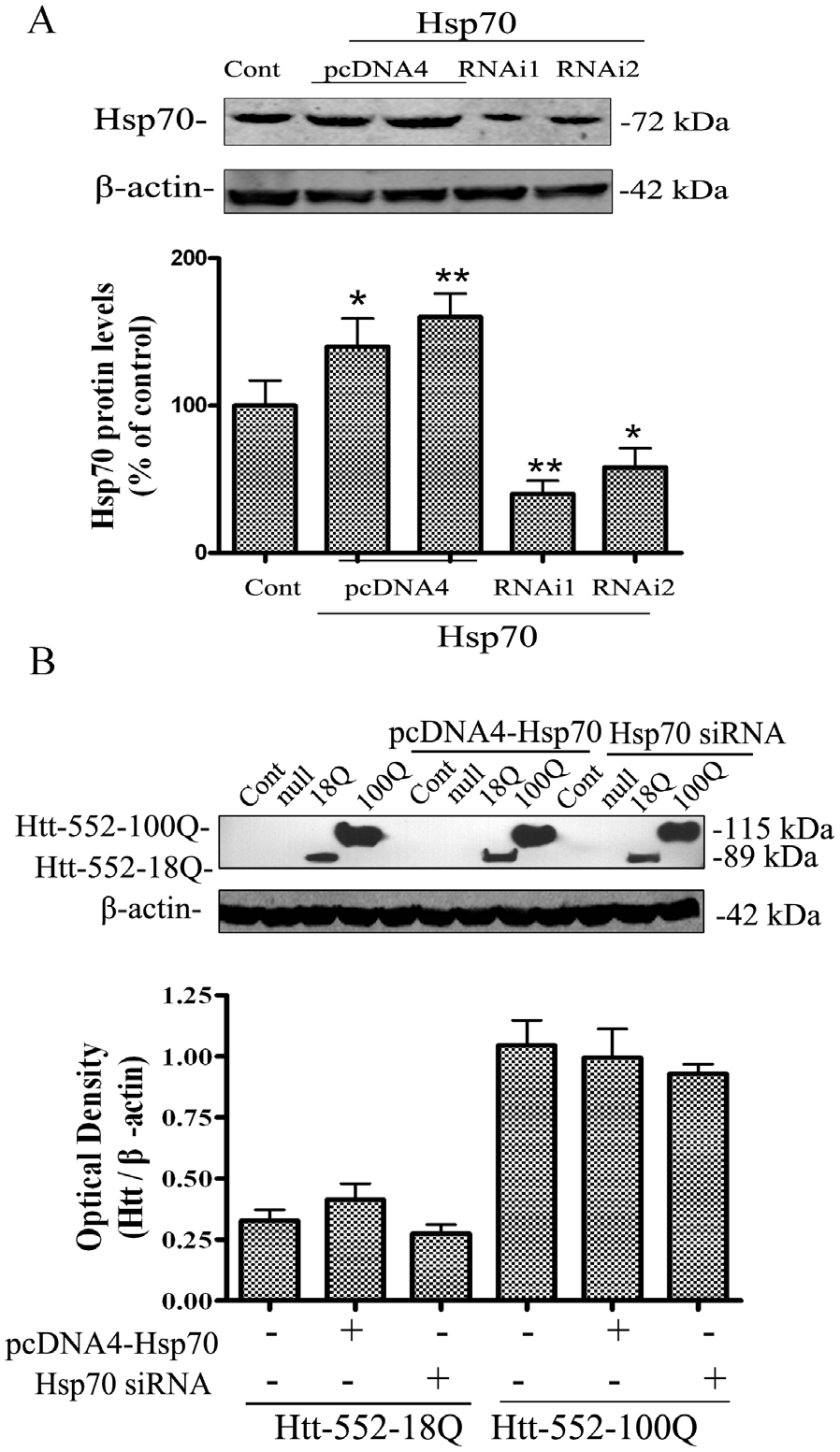
Hsp70 had no affect the clearance of Htt-552. (A) The efficiency of knockdown and overexpression of Hsp. HeLa cells were transiently transfected with Hsp70 plasmid or Hsp70 siRNA for 72 h. Lysates were subjected to Western blot analysis with anti-Hsp70 antibody. Values are the mean±SE of three independent experiments. *P<0.05, **P<0.01 (vs control). (B) Hsp70 had no effect on Htt-552 levels. HeLa cells were transiently transfected with Hsp70 plasmid, vector control or Hsp70 siRNA for 72 h and Htt-552 for 48 h. Lysates were subjected to Western blot analysis with anti-Htt 2166 and anti–Hsp70 antibodies. Values are the mean±SE of three independent experiments.

To assess the role of CMA in degradation of Htt-552, the present study examined the effects of increasing and decreasing the levels of LAMP-2A or Hsc70 on Htt-552 accumulation. Increased peri-nuclear localization of cathepsin D-positive lysosomes was observed in the cells after exogenous expression of LAMP-2A or Hsc70 (**Figure 3A and 3E**). The effectiveness of exogenous expression and knockdown of LAMP-2A and Hsc70 was confirmed with Western blot analysis (**Figure 3B and 3F**). There was an increase in levels of Htt-552-18Q and Htt-552-100Q when endogenous LAMP-2A was knocked down with siRNA. In contrast, overexpression of LAMP-2A decreased the accumulation of both wt and mutant Htt-552 (**Figure 3C and 3D**). It was noticeable that the magnitude of changes was more robust for levels of Htt-552-18Q than Htt-552-100Q. Similarly, knockdown of Hsc70 with siRNA increased accumulation of Htt-552, while overexpression of Hsc70 decreased the accumulation of Htt-552 (**Figure 3G**). In contrast, change in Hsp70 protein levels had no significant effect on accumulation of Htt-552 (**Figure 4A and 4B**). These data are consistent with a role for LAMP-2A and Hsc70 in CMA dependent removal of Htt.

### Uptake of Htt-552 by lysosomes

**Figure 5:**
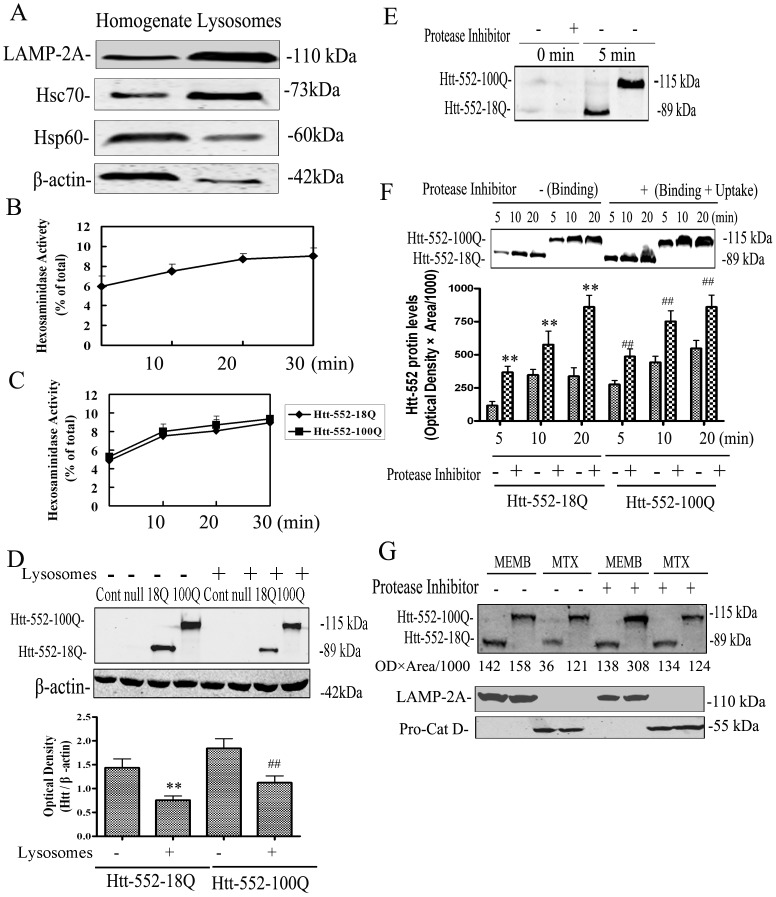
Degradation of Htt-552 by lysosomes. (A) The purity of the isolated lysosomes. Total cellular homogenates and lysosomal fraction (30 µg of proteins) were subjected to SDS-PAGE and immunoblotting for the indicated proteins. Actin was shown as a marker of cytoplasm. Hsp60 was shown as a marker of mitochondrial. LAMP-2A and Hsc70 were shown as markers of lysosomes. (B) The integrity of the isolated lysosomes. The release of β-hexosaminidase from lysosomes was indicated with incubation of lysosomes with para-nitrophenylphosphate (PNPP). The reaction was terminated with 0.25 mol/L NaOH. Release of all lysosomal β-hexosaminidase was induced by incubation with 0.1% Triton X-100. Release of all lysosomal β-hexosaminidase was induced by incubation with 0.1% Triton X-100. Values of β-hexosaminidase activity are expressed as percentage of total activity in the lysosomal fraction at time 0, 10, 20 and 30 min and are means±SE of three independent experiments. (C) Effects of Htt-552 on lysosomal stability. The release of β-hexosaminidase from intact lysosomes incubated with Htt-552-18Q or Htt-552-100Q. PC12 cells were transfected with Htt-552 for 48 h. Cell lysates were used as Htt-552-riched lysates. The lysates containing Htt-552 were incubated with isolated lysosomes and the lysosomal β-hexosaminidase activity at time 0, 10, 20 and 30 min was determined. Release of all lysosomal β-hexosaminidase from the same batches of lysosomes was induced by incubation with 0.1% Triton X-100. Release of all lysosomal β-hexosaminidase from the same batches of lysosomes was induced by incubation with 0.1% Triton X-100. The values are means±SE of three independent experiments. (D) Effects of lysosomes on the degradation of Htt-552. PC12 cells were trasfected with Htt-552 as described above and cell lysates were incubated with isolated lysosomes. The degradation of Htt-552-18Q and Htt-552-100Q after incubating with intact lysosomes was assessed with Western blot analysis. Values are the mean±SE of three independent experiments. **P<0.01 (compared with Htt-552-18Q without treatment); ^##^P<0.01 (compared with Htt-552-100Q without treatment). (E) Association of increasing Htt-552 with isolated lysosomes untreated (Binding) or pre-treated with protease inhibitors (Binding+Uptake) at 0 and 5 min. Values are the mean±SE of three independent experiments. (F) Association of increasing Htt-552 with isolated lysosomes untreated (Binding) or pre-treated with protease inhibitors (Binding+Uptake) at 5, 10 and 20 min. Values are the mean±SE of three independent experiments. **P<0.01 (compared with Htt-552-18Q without treatment); ##P<0.01 (compared with Htt-552-100Q without treatment). (G) Htt-552 in lysosomal membranes (MEMB) and matrices (MTX). Isolated lysosomes were incubated cell lysates containing expressed Htt-552 for 20 min, lysosomes were recovered by centrifugation. Lysosomal membranes and matrix were separated after hypotonic shock and centrifugation, and were processed for Western blot analysis.

The most direct evidence for a protein as a CMA substrate probably is to determine its binding, uptake, and degradation in isolated intact lysosomes [17]. The purity of the lysosome preparation was detected by the enrichment of the lysosomal markers LAMP-2A and Hsc70 and a marked decrease in levels of Hsp60, which is a marker of mitochondria, and in β-actin, which is a marker of cytoplasm. Based on these assessments, the present study had achieved isolation of lysosomes from mouse liver with a relatively high purity (**Figure 5A**). β-hexosaminidase latency was measured as an index of the integrity of lysosomal membranes after isolation [38]. Results indicated that the percent of broken lysosome content was 6.1+0.3% of total at 0 min, 7.8+0.4% of total at 10 min, 9.2+0.3% of total at 20 min and 9.4+0.4% of total at 30 min (**Figure 5B**), indicating that the lysosomes in the preparation remained mostly intact.

Disease-causing mutant proteins had been suggested to destabilize the membranes of lysosomes [39]. Therefore we first confirmed that isolated lysosomes were not disrupted by either wt or mutant Htt-552 proteins by detecting the leakage of β-hexosaminidase from lysosomes after incubation with cell lysates containing exogenously expressed Htt-552-18Q or Htt-552-100Q (**Figure 5C**). Incubation of cell lysates with lysosomal preparations significantly reduced the protein levels of Htt-552, reflecting degradation of Htt-552 by lysosomes (**Figure 5D**). Next, we studied the association of Htt-552 to lysosomal membranes and uptake of Htt-552 into lysosomal lumen. Htt-552 was recovered in the absence of protease inhibitors, indicating its presence in the lysosomal membranes; Htt-552 was also recovered in the presence of protease inhibitors, indicating its localization to the lysosomal lumen and to membranes (**Figure 5E**). Detection of Htt-552 binding to lysosomal membranes (no protease inhibitor) increased with incubation time. The level of lysosomal membrane bound Htt-552 was higher in the presence of the protease inhibitor cocktail. Moreover, the magnitude of increases in Htt-552-18Q was more robust than that of Htt-552-100Q (**Figure 5F**). The difference of lysosomal Htt-552 in the presence and absence of protease inhibitors represents the amount of Htt-552 taken up by lysosomes.

After disrupting the lysosomes and separating lysosomal membranes and matrix fractions [34], Htt-552 was detected in both membrane and matrix fractions, suggesting that Htt-552 translocated into the lysosomal lumen. Cathepsin D is a luminal marker and LAMP-2A is a lysosomal membrane marker [40]. Inhibition of lysosomal proteases increased the accumulation of Htt-552-100Q in lysosomal membranes, however the membrane Htt-552-18Q levels were not significantly changed (**Figure 5G**). In contrast, the accumulation of Htt-552-18Q but not Htt-552-100Q in the lysosomal matrix was observed in the presence of protease inhibitors, indicating that Htt-552-18Q is more readily taken into lysosomes for degradation than is Htt-552-100Q (**Figure 5F**).

**Figure 6:**
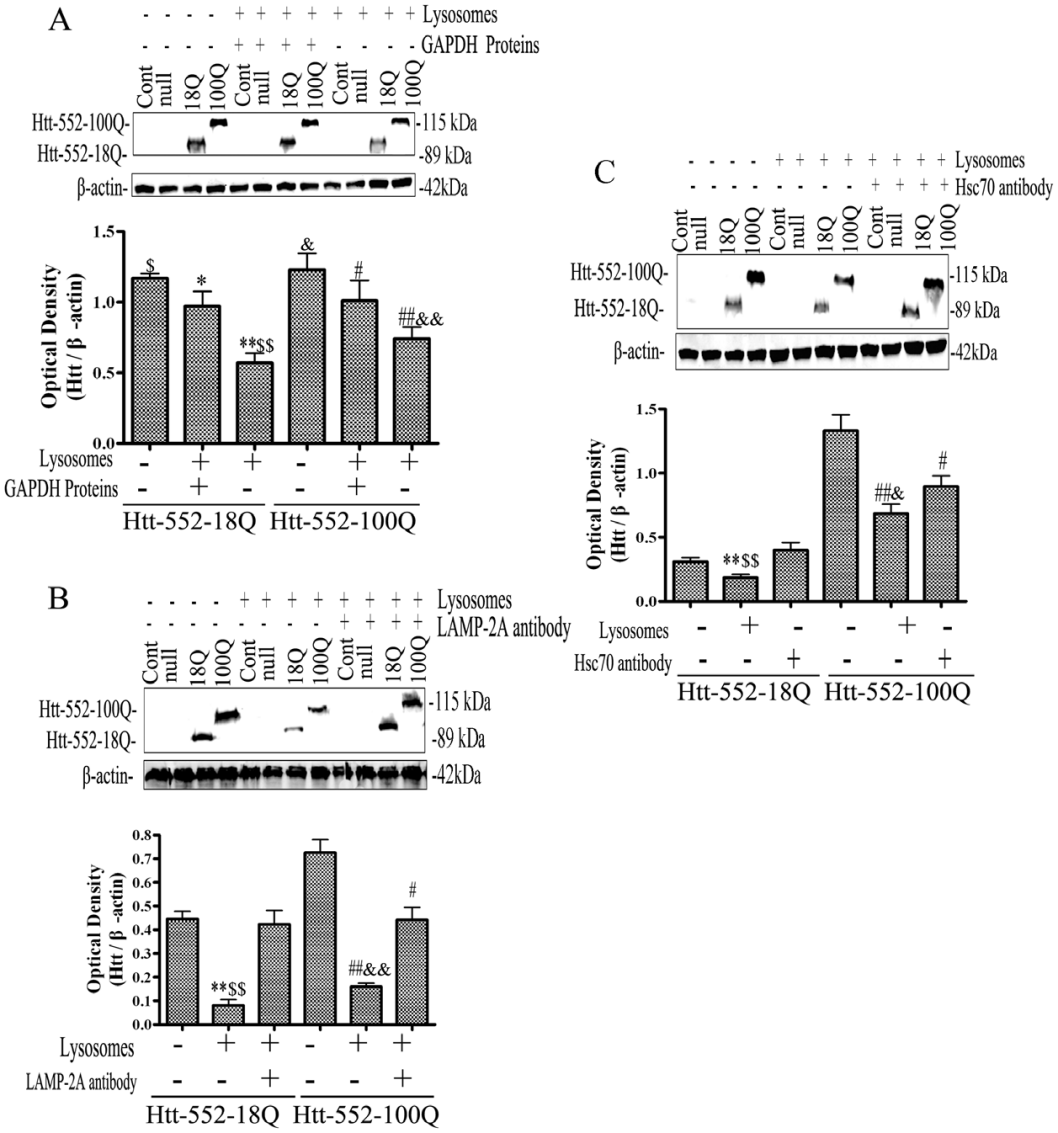
Inhibition of Htt-552 degradation by blocking CMA pathway. (A) The effects of GAPDH on degradation of Htt-552. The isolated lysosomes were incubated with 10 µg GAPDH and Htt-552 for 30 min at 37°C. At the end of the incubation, lysosomes were collected by centrifugation and the levels Htt-552 were determined with immunoblotting. Values are the mean±SE of three experiments. **P<0.01, *P<0.05 (compared with Htt-552-18Q without lysosomes); ^##^P<0.01, ^#^P<0.05 (compared with Htt-552-100Q without lysosomes); ^$$^P<0.01 (compared with Htt-552-18Q without GAPDH); ^&&^P<0.01 (compared with Htt-552-100Q without GAPDH). (B) The effects of LAMP-2A neutralizing antibody on degradation of Htt-552 by isolated lysosomes. The incubation of lysosomal preparation with Htt-552 was described as above. Values are the mean±SE of three independent experiments **P<0.01 (compared with Htt-552-18Q without lysosomes); ^#^P<0.05, ^##^P<0.01 (compared with Htt-552-100Q without lysosomes); ^$$^P<0.01 (compared with Htt-552-18Q without LAMP-2A antibody); ^&&^P<0.01 (compared with Htt-552-100Q without LAMP-2A antibody). (C) The incubation of lysosomal preparation with Htt-552 was described as above. The incubation of lysosomal preparation with Htt-552 was described as above. The effects of Hsc70 neutralizing antibody on degradation of Htt-552 by isolated lysosomes. Values are the mean±SE of three independent experiments. **P<0.01 (compared with Htt-552-18Q without lysosomes); ^##^P<0.01, ^#^P<0.05 (compared with Htt-552-100Q without lysosomes); ^$$^P<0.01 (compared with Htt-552-18Q without Hsc70 antibody); ^&^P<0.05 (compared with Htt-552-100Q without Hsc70 antibody).

As CMA substrates use LAMP-2A and Hsc70 for recognition and membrane transport, there is a competition between CMA substrates for these mediators. Glyceraldehyde-3-phosphate dehydrogenase (GAPDH) is a known CMA substrate. This study evaluated the effects of Htt-552 degradation by GADPH. The results showed that pre-incubation of isolated lysosomes with GAPDH for 30 min reduced degradation of Htt-552 by lysosomes (**Figure 6A**). Furthermore, pre-incubation of isolated lysosomes with neutralizing antibodies against LAMP-2A and Hsc70 also decreased the degradation of Htt-552 by lysosomes (**Figure 6B and 6C**).

### Disruption of interactions between Htt-552 and Hsc70 by mutation of CMA recognition motifs

**Figure 7:**
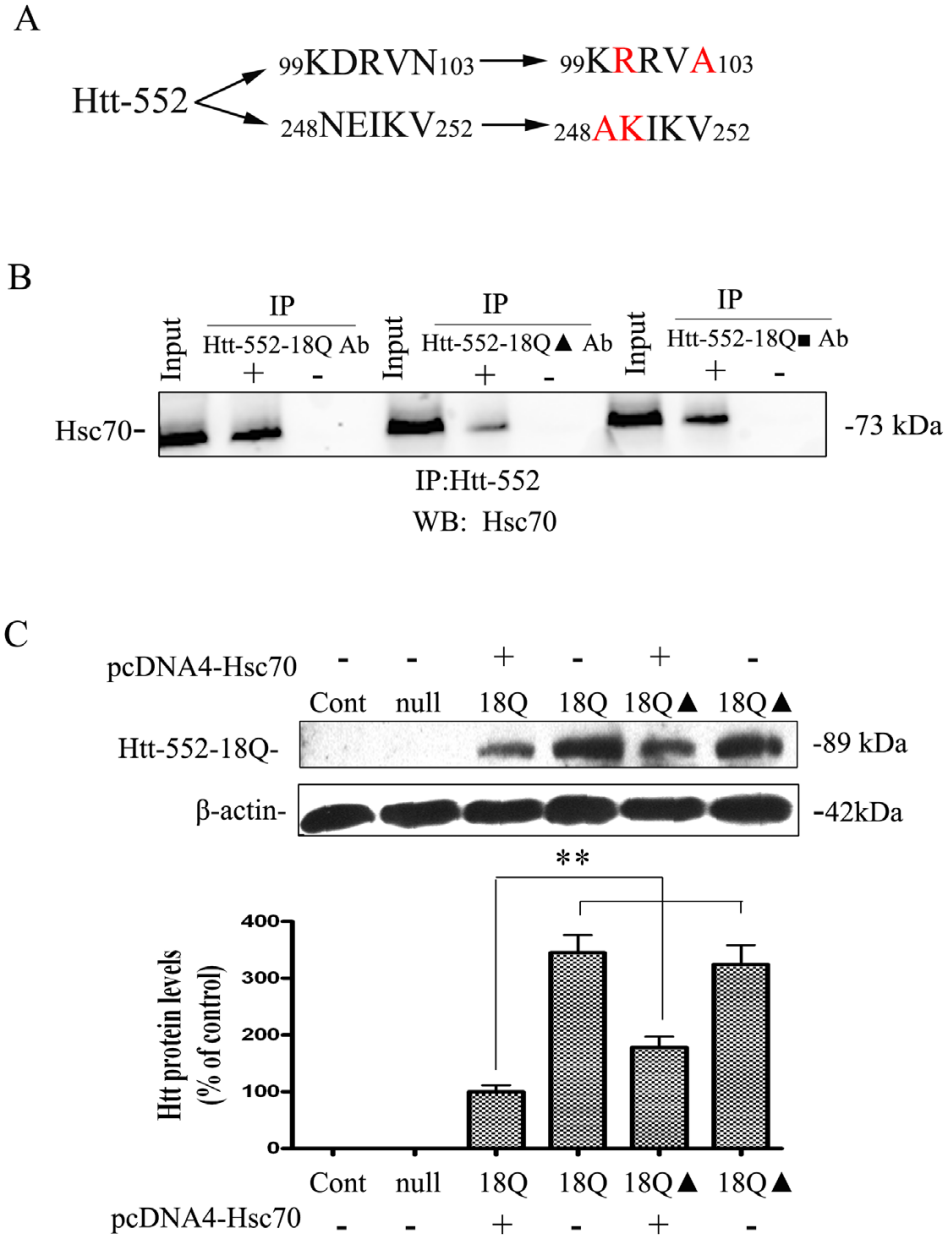
The effects of mutation of recognition motifs in wt Htt-552 on Htt-552 degradation. (A) Mutation of Htt-552 of 18Q at 99KDRVN103 and 248NEIKV252. (B) Immunoprecipitation of exogenous expressed Hsc70 and Htt-552-18Q. Hella cells were transfected with wt and mutant Htt-18Q for 48 h. The lysates were subjected to immunoprecipitation with anti-Hsc70 antibody or no primary antibody. Mutation of Htt-552-18Q at 99-103 was labeled with ▴ and mutation of Htt-552-18Q at 248-252 was labeled with ▪. Ab: Antibody. (C) Inhibition of degradation of Htt-552-18Q with mutation of CMA recognition sites. The degradation of Htt-552-18Q in HeLa cells transiently transfected with human Hsc70 plasmid or vector control and Htt-552-18Q▴ was determined with Western blot analysis. Values are the mean±SE of three independent experiments. **P<0.01 (compared with the expression of Htt-552-18Q without treatment).

We mutated the sequence _99_KDRVN_103_ and _248_NEIKV_252_ in a putative CMA recognition motif in Htt-552-18Q by replacing hydrophilic amino acid asparagine (N) with hydrophobic alanine (A), replacing acidic aspartate (D) with basic arginine (R) of _99_KDRVN_103_ (Htt-552 of 18Q▴) and replacing glutamine (E) with basic lysine (K) of _248_NEIKV_252_ (Htt-552 of 18Q▪) (**Figure 7A**). The results showed that although the transfection efficiency of adenovirus Htt-552-18Q with the mutant recognition motif (Htt-552 of 18Q▴) in PC12 cells was similar to wild-type Htt-552-18Q, the co-precipitation of mutant Htt-552-18Q▴ protein with Hsc70 was reduced compared with the wt Htt-552-18Q (**Figure 7B**). However the co-precipitation of mutant Htt-552 -18Q▪ protein with Hsc70 was not dramatically changed compared with that of Htt-552-18Q. Overexpression of human Hsc70 markedly decreased accumulation of mutant Htt-552. However, the overexpression of Hsc70 was less effective in reducing Htt-552-18Q▴ in comparison with Htt-552, suggesting that the clearance of Htt-552-18Q▴ may be inhibited by the mutation of the CMA recognition motif (**Figure 7C**). The data suggest that _99_KDRVN_103_ of Htt-552 is a functional site for substrate recognition by CMA.

### CMA affects degradation of endogenous Htt

**Figure 8:**
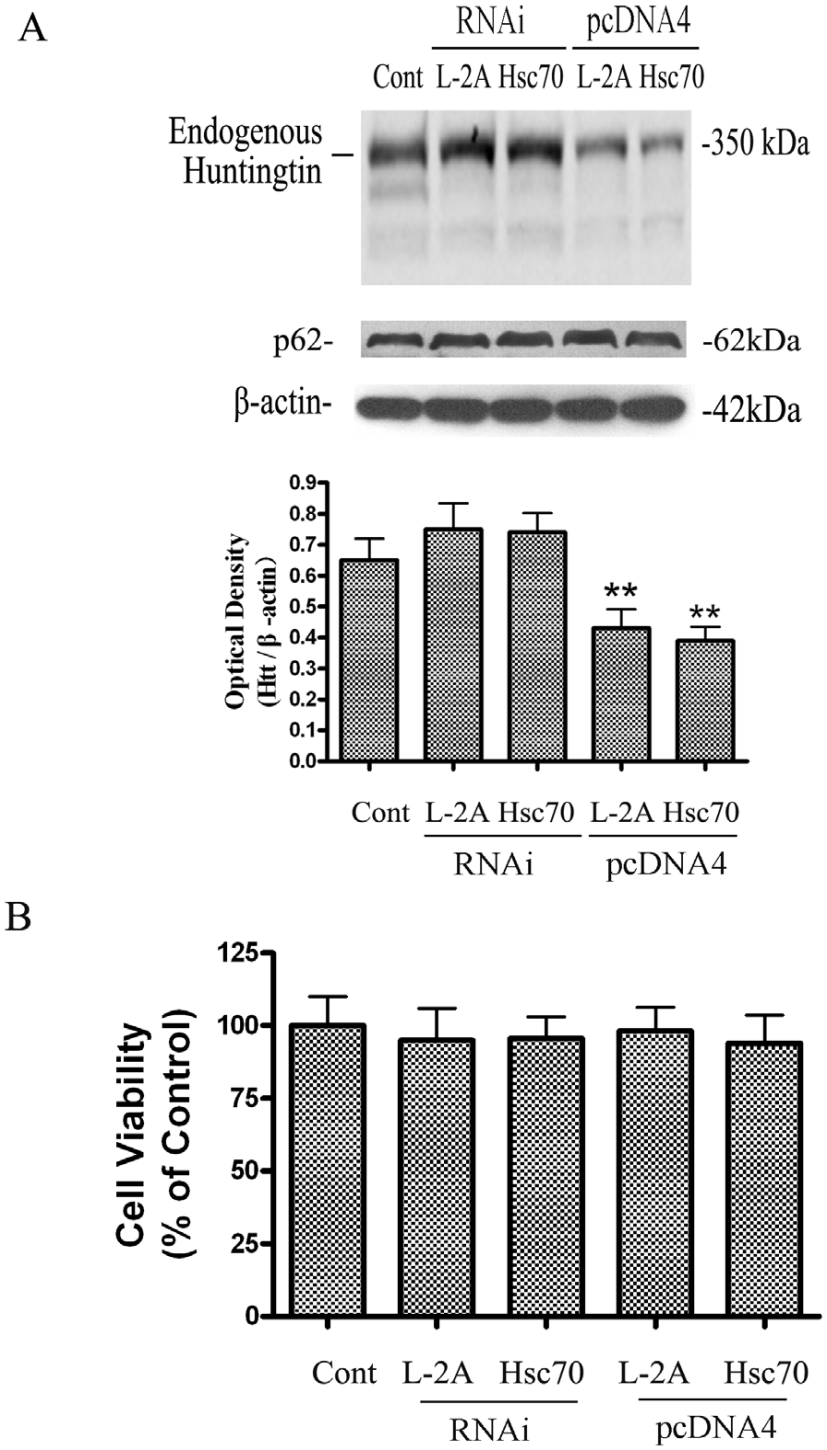
The effects of CMA on degradation of endogenous Htt. (A) The effects of knockdown and overexpression of LAMP-2A and Hsc70 on endogenous Htt and p62. PC12 cells were transiently transfected with LAMP-2A, Hsc70 plasmid, vector control or LAMP-2A and Hsc70 siRNA for 48 h. Lysates were subjected to Western blot analysis with antibodies against Htt and p62. Values are the mean±SE of three independent experiments. **P<0.01 (compared with endogenous Htt without treatment). (B) The PC12 cell viability following change of CMA. Following knockdown or overexpression of LAMP-2A and Hsc70 for 48 h, cell viability was analyzed with the MTT assay. Values were given as mean ± SD of three independent experiments.

To verify the role of CMA in degradation of endogenous Htt, the effects of reducing and increasing LAMP-2A on endogenous Htt were determined. Endogenous Htt was significantly reduced in PC12 cells that overexpressed LAMP-2A or Hsc70 for two days and was slightly increased (though not statistically significant) in PC12 cells treated with LAMP-2A or Hsc70 siRNA for two days (**Figure 8A**). The reduction in endogenous Htt after expression of LAMP-2A or Hsc70 was not due to general depression of protein synthesis as no change was detected in the levels of β-actin and p62. The later is a substrate of macroautophagy but not CMA (**Figure 8A**). The decrease in endogenous Htt was also not due to loss of cell viability as the level of MTT transformation was not different between control untreated cultures and those exposed to cDNAs encoding LAMP-2A or Hsc70 (**Figure 8B**).

## Discussion

HD is a family of polyQ repeat expansion diseases characterized by the accumulation of mutant Htt protein in diseased neurons [1]. The present study used the first 552 amino acids of human Htt with an expanded polyglutamine tract to simulate cellular pathologic conditions of the disease because a native Htt fragment of this size has been detected in vivo in human brain and in HD animal models [41], [42]. Moreover this N-terminal region of Htt contains domains and modifications that regulate the targeting and turnover of the full-length protein [26]. Expressed Htt-552 was detected by a well-described anti-Htt antibody, MAB2166 which recognizes Htt443-457. There are reports that MAB2166 may not interact well with Htt species that have been modified by phosphorylation or acetylation which may be important for its degradation [26]. To assure the accuracy of Htt-552 detection, we also used an antibody recognizing Htt1-17 and obtained similar results related to CMA dependent clearance (**Figure S2 A**).

Autophagy is an important intra-cellular metabolic pathway that includes macroautophagy, microautophagy and CMA. All of the substrates of autophagy are transported to lysosomes where the substrates are degraded by hydrolytic enzymes. Blockade of lysosomes means blockade of all three types of autophagic degradation. The best characterized autophagy is macroautophagy which probably contributes to the highest percentage of lysosomal degradation in cells [43]. There are reports that the accumulation of Htt is decreased through the activation of lysosomes [4], [7] whereas Htt accumulates with the inhibition of autophagy [5], [25]. The results of previous studies indicate that Htt-552 is not only degraded by macroautophagy but also by CMA. Although involvement of CMA in Htt metabolism has been suggested by other investigators, the essential evidence of CMA metabolism of Htt, including interactions of Htt with LAMP-2A, Hsc70, and uptake and degradation of Htt by lysosomes remains to be revealed. Thus, this work presented direct evidence for huntingtin clearance by CMA.

It is known that after serum removal, activation of macroautophagy occurs before the activation of CMA, reaches maximal activity 4–6 h after starvation, and then gradually declines to basal levels. In most cells continuous starvation beyond 6 h, will increase CMA activity to maximal levels at about 12 h [44] and continue as long as starvation persists. The time-course of induction of Beclin 1 and LAMP-2A was consistent with the activation of macroautophagy and CMA with different time frames. The activation of CMA with serum removal was supported by the presence of increased localization of lysosomes to peri-nuclear regions [37]. We found decreased levels of exogenous Htt-552 at 6 h (macroautophagy activation reached peak level) and 12 h (macroautophagy returned to basal level and CMA activation reached peak level) after starvation. These results suggest that both macroautophagy and CMA are involved in degradation of Htt-552. We also observed more Htt-552 clearance by macroautophagy than by CMA. However, due to its greater selectivity for substrates CMA may be more important than macroautophagy in degrading Htt.

To further assess the association between Htt-552 and CMA, this study observed the interactions of exogenous expressed Htt-552 with the critical components of CMA, LAMP-2A and Hsc70. LAMP-2A is the integral membrane receptor protein that can directly import CMA substrate proteins across the lysosomal membranes. Hsc70 is a CMA chaperone which preferentially interacts with CMA substrate proteins and assists proteins moving into lysosomal lumens [19]. Immunofluorescence data indicated that Htt-552 co-localized with LAMP-2A and Hsc70. Immunoprecipitation results confirmed that Htt-552 could interact with Hsc70 and LAMP-2A. However, co-immunoprecipitation of Htt-552 with LAMP-2A was not efficient, especially for wt. The possible reason could be that LAMP-2A is a membrane protein and is not easy to be precipitated with current protocol. It was noticed that mutant Htt-552 had stronger interactions with the critical components of CMA than did wt Htt-552. To assess the role of Hsc70 and LAMP-2A in mediating Htt-552 degradation, alterations in LAMP-2A and Hsc70 protein levels were achieved with adenovirus mediated overexpression or siRNA technology. Regulation of LAMP-2A and Hsc70 levels appeared to affect Htt-552 degradation in cells, presumably by changing CMA activity. Although Hsp70 is highly homologous to Hsc70 and almost indistinguishable from it in chaperone activity [43], the manipulation of Hsp70 levels had no significant effect on Htt-552 accumulation. These data suggest that Htt is degraded by a LAMP-2A and Hsc70–dependent mechanism through CMA.

In vivo, both macroautophagy and CMA use lysosomes for substrate degradation and differ in the way substrates are delivered into lysosomes. Macroautophagy contributes to the highest percentage of lysosomal degradation inside cells. The cytosolic components must be sequestered inside the autophagosomes to be degraded upon fusion of the membrane of autophagosomes with lysosomes [7], [8]. Microautophagy is well-studied in yeast, but the understanding of microautophagy in mammals is limited because mammalian homologs of the yeast genes have not been identified [16], [18], [21]. For CMA degradation, proteins are directly taken up by lysosomal membranes through LAMP-2A. Determining a protein's binding, uptake, and degradation in isolated intact lysosomes is the most direct test whether a protein is a CMA substrate [19], [20], [45], [46]. Thus, it was critical to demonstrate in our study that Htt-552 is taken up by lysosomes. We successfully isolated intact lysosomes from mouse liver that were suitable for studying lysosomal uptake of Htt-552 [37]. Because the lysosomal stabilization may be affected by some proteins [39], we first confirmed that isolated lysosomes were not disrupted by either wt Htt-552 or mutant Htt-552. The translocation of Htt-552 into lysosomal lumen and degradation by lysosomal enzymes were confirmed by the following evidence: 1 addition of isolated lysosomes to cell lysates containing exogenously expressed Htt-552 robustly reduced Htt-552 levels; 2 in the above assay system, LAMP-2A and Hsc70 neutralizing antibodies inhibited the lysosome-mediated Htt-552 degradation; 3 CMA substrate GAPDH [20], [45] reduced the lysosome-mediated Htt-552 degradation by competing for LAMP-2A; 4 sub-lysosomal fractionation [37] revealed Htt-552 in the lysosomal membranes and matrix. In addition, this study found that after inhibiting lysosomal degradation, more mutant Htt-552 was accumulated in the lysosomal membranes but little change occurred in the matrix. In contrast, more wt Htt-552 was accumulated in the matrix but little change happened in the lysosomal membranes. In this study, the differences in effects in CMA activity on Htt-552-18Q and Htt-552-100Q were noticeable. The change in Htt-552-18Q accumulation was bigger than that of Htt-552-100Q when CMA was inhibited by inhibitors or knockdown of LAMP-2A and Hsc70; or when CMA was activated by starvation or overexpression of functional proteins of CMA. In isolated lysosomes, mutant Htt-552 was less able than wt Htt-552 to cross lysosomal membranes and be degraded by lysosomal enzymes. These data suggest that expansion of a polyQ tract impairs Htt clearance through CMA.

All substrate proteins of CMA contain a motif consisting of the pentapeptide KFERQ that targets the proteins to lysosomes [47]. This motif is recognized by Hsc70, which interacts with the substrate proteins in the cytosol [33], [48]. The putative KFERQ motif in Htt-552 was analyzed as described by Liang et al [49]. There are two KFERQ-like motifs found between amino acid 99-103 (_99_KDRVN_103_) and 248-252 (_248_NEIKV_252_) [47]. The amino acid sequence _14_LKSFQ_18_ is also considered to be a KFERQ-like motif when it is phosphorylated [26]. Mutation of CMA recognition motif was achieved by changing hydrophilic and hydrophobic amino acids in KFERQ-like motif located between aa99-103 and 248-252. The results showed that mutation of amino acids in 99-103 but not in 248-252 reduced Htt-552 interaction with Hsc70 and also reduced clearance of Htt-552. These data suggest that a KFERQ-like motif located between aa99-103 is a functional site for recognition by Hsc70. Due to technical difficulty, mutating these sites in Htt-552-100Q was unsuccessful. Some studies have suggested degradation of Htt by CMA. For example, Thompson et al verified that phosphorylated Htt at S13 could form a KFERQ-like motif [26]. Furthermore, Bauer et al found that the degradation of Htt fragment could be increased by Hsc70 [25]. The present study identified a new functional putative KFERQ motif in Htt-552 that bound Htt-552 to Hsc70 and allowed uptake by lysosomes via LAMP-2A.

In summary, this study provides evidence elucidating a role of CMA in degradation of Htt-552. Expansion of the polyQ tract in Htt may slow its transport across lysosomal membranes and thus increase its accumulation in the cytosol. The confirmation of the involvement of the CMA pathway in Htt clearance is important, as this pathway is relatively selective for its substrates. Thus developing therapies that target an enhancement of CMA-mediated clearance of mutant Htt may have great value.

## Supporting Information

Figure S1 (A) Expression of Htt-552-18Q/100Q in PC12 or HeLa cells after infection with adenoviral vectors. Immunofluorescence analysis was carried out 48 h after infection. Htt-552-18Q and 100Q were labeled in red with anti-2166 antibody, while the DAPI identified the cell nuclei in blue. Cells were analyzed using confocal microscopy. (B) Effects of adenoviral vectors expressing Htt-552 on viability of PC12 or HeLa cells. Following exposure of PC12 or HeLa cells to various concentrations of virus vectors, cell viability was analyzed by the MTT assay 48 h post-infection. Values were given as mean ± SD of 3 independent experiments. Null: infected with Ad-null; 18Q: infected with Ad-Htt-552-18Q; 100Q: infected with Ad-Htt-552-100Q (m.o.i. of 210). (C) Death rate of PC12 or HeLa cells was analyzed by the LDH assay 48 h post-infection. Values were given as mean ± SD of 3 independent experiments. Null: infected with Ad-null; 18Q: infected with Ad-Htt-552-18Q; 100Q: infected with Ad-Htt-552-100Q (m.o.i. of 210).
(TIF)

Figure S2 (A) Detection of the expression of Htt-552-18Q/100Q in PC12 cells after infection with adenoviral vectors with MAB2166 or Ab1 antibodies with Western Blot analysis. Values are the mean±SE of three independent experiments. (B) Effects of expression of Htt-552 on cell viability following autophagy inhibition (48 h). Following exposure of PC12 cells to various concentrations of virus vectors, cell viability was analyzed by the MTT assay at 48 h post-infection. Values were given as mean ± SD of 3 independent experiments. Null: infected with Ad-null; 18Q: infected with Ad-Htt-552-18Q; 100Q: infected with Ad-Htt-552-100Q (m.o.i. of 210). (C) Effects of expression of Htt-552 on cell viability following siRNA of LAMP-2A and Hsc70 (120 h). Following exposure of PC12 cells to virus vectors, cell viability was analyzed by the MTT assay at 120 h post-infection. Values were given as mean ± SD of 3 independent experiments. Null: infected with Ad-null; 18Q: infected with Ad-Htt-552-18Q; 100Q: infected with Ad-Htt-552-100Q (m.o.i. of 210). **P<0.01,*P<0.05 (compared with Htt without treatment). ##P<0.01, #P<0.05 (Htt-552-100Q compared with Htt-552-18Q). (D) Effects of expression of Htt-552 on cell cycle distribution following treatment with siRNA targeting LAMP-2A or Hsc70 (120 h). The cell cycle analysis and cell apoptosis were determined using FACS analysis as described in Methods and Materials. ,*P<0.05 (compared with Htt without treatment). ##*P*<0.01, #*P*<0.05 (Htt-552-100Q compared with Htt-552-18Q).
(TIF)

Figure S3 **Association of Htt-552 and endogenous CMA component proteins.** (A) Endogenous expressed LAMP-2A or Hsc70 co-localized with exogenous Htt-552-100Q in HeLa cells. Cells were transfected with Htt-552 for 48 h and fixed with methanol, blocked, and processed for double immunofluorescence with antibodies against Htt-552-100Q (red) Htt-552-100Q (red) and LAMP-2A (green) or Hsc70 (green). Merged images of both channels are shown at right. N: nucleus. Thin arrows point to Htt-552 immunoreactivity. Thick arrows point to lysosomes. The scale bar = 10 µm. (B) Co-immunoprecipitation of endogenous expressed LAMP-2A with exogenously expressed Htt-552-100Q. Upper panel: Cell lysates were subjected to immunoprecipitation with anti-Htt antibody or no primary antibody and Western blot analysis was performed with anti-LAMP-2A antibody. Lower panel: PC12 cell were transfected with Htt-552-100Q for 48 h. Cell lysates were subjected to immunoprecipitation with anti-LAMP-2A antibody or no primary antibody and Western blot analysis was performed with anti-Htt antibody. (C) Co-immunoprecipitation of endogenous expressed Hsc70 with exogenously expressed Htt-552-100Q. Upper panel: Cell lysates were subjected to immunoprecipitation with anti-Hsc70 antibody or no primary antibody and Western blot analysis was performed with anti-Htt antibody. Lower panels:PC12 cell were transfected with Htt-552-100Q for 48 h. Cell lysates were subjected to immunoprecipitation with anti-Htt antibody or no primary antibody and Western blot analysis was performed with anti-Hsc70 antibody. Ab: Antibody.
(TIF)
