# Supplementary figures and images for: The Role of Chaperone-Mediated Autophagy in Huntingtin Degradation
Source: PLoS One. 2012 Oct 11;7(10):e46834. doi: 10.1371/journal.pone.0046834 (PMC3469570; doi:10.1371/journal.pone.0046834)

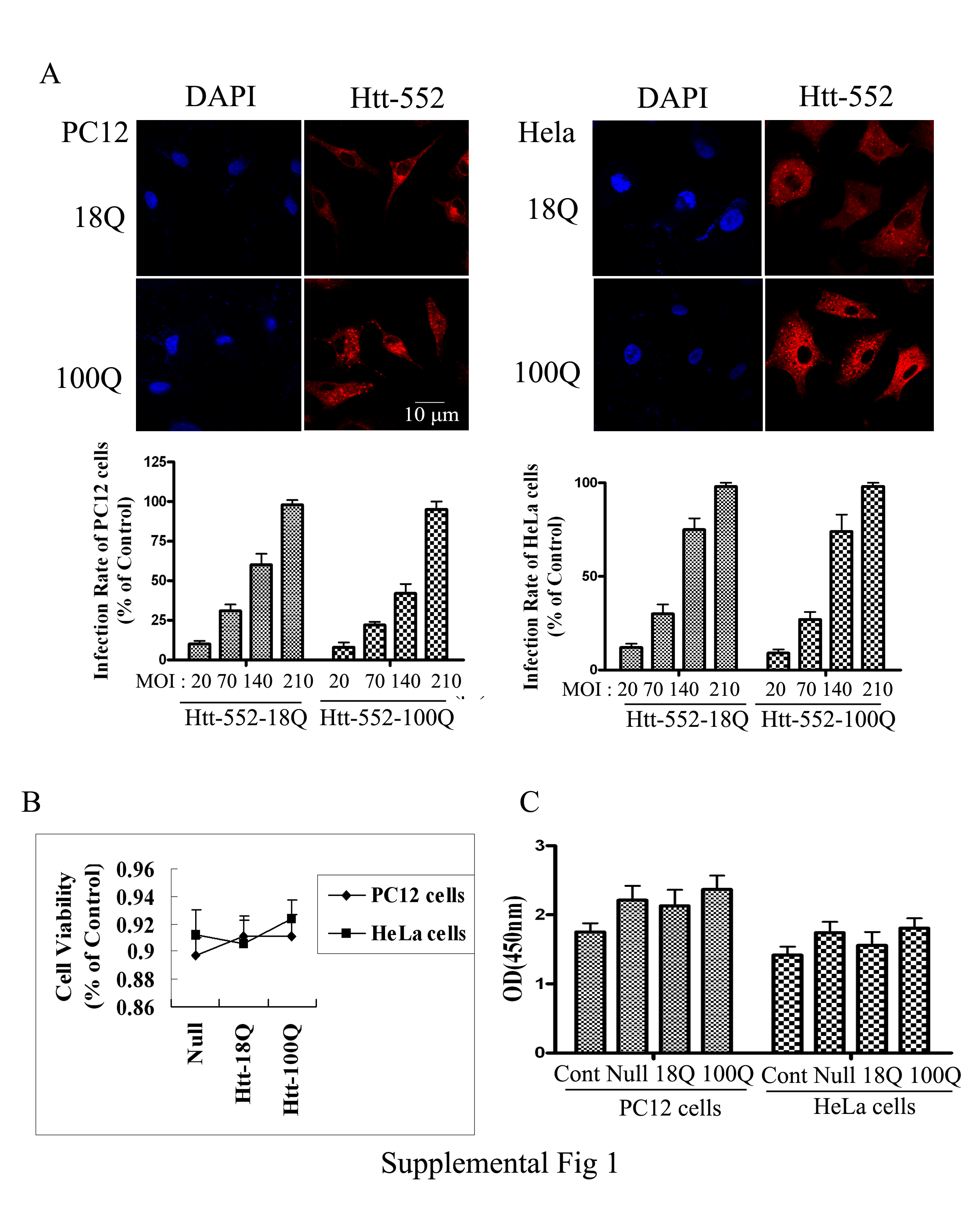

Supplement: Figure S1 — (A) Expression of Htt-552-18Q/100Q in PC12 or HeLa cells after infection with adenoviral vectors. Immunofluorescence analysis was carried out 48 h after infection. Htt-552-18Q and 100Q were labeled in red with anti-2166 antibody, while the DAPI identified the cell nuclei in blue. Cells were analyzed using confocal microscopy. (B) Effects of adenoviral vectors expressing Htt-552 on viability of PC12 or HeLa cells. Following exposure of PC12 or HeLa cells to various concentrations of virus vectors, cell viability was analyzed by the MTT assay 48 h post-infection. Values were given as mean ± SD of 3 independent experiments. Null: infected with Ad-null; 18Q: infected with Ad-Htt-552-18Q; 100Q: infected with Ad-Htt-552-100Q (m.o.i. of 210). (C) Death rate of PC12 or HeLa cells was analyzed by the LDH assay 48 h post-infection. Values were given as mean ± SD of 3 independent experiments. Null: infected with Ad-null; 18Q: infected with Ad-Htt-552-18Q; 100Q: infected with Ad-Htt-552-100Q (m.o.i. of 210). (TIF) [file pone.0046834.s001.tif]

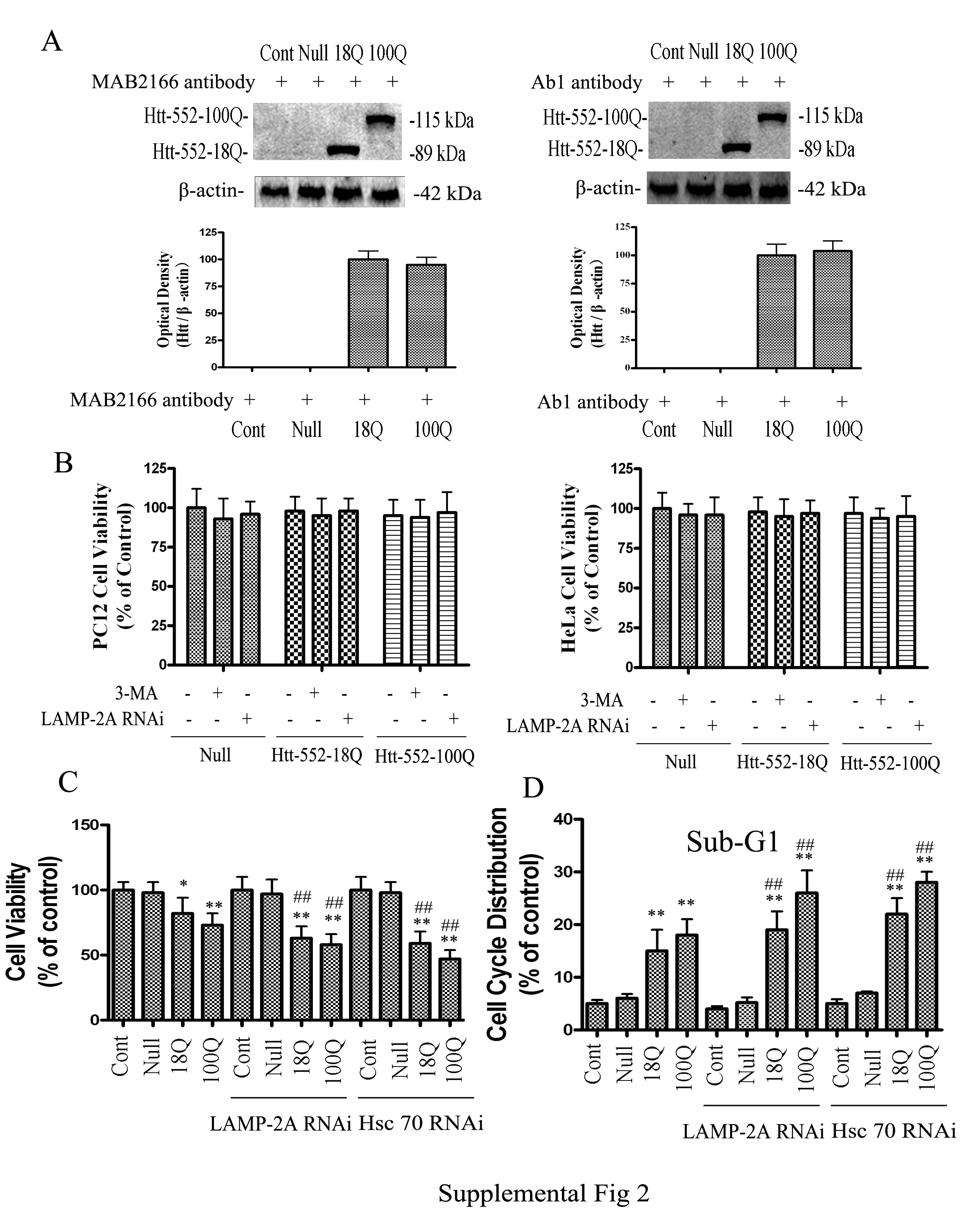

Supplement: Figure S2 — (A) Detection of the expression of Htt-552-18Q/100Q in PC12 cells after infection with adenoviral vectors with MAB2166 or Ab1 antibodies with Western Blot analysis. Values are the mean±SE of three independent experiments. (B) Effects of expression of Htt-552 on cell viability following autophagy inhibition (48 h). Following exposure of PC12 cells to various concentrations of virus vectors, cell viability was analyzed by the MTT assay at 48 h post-infection. Values were given as mean ± SD of 3 independent experiments. Null: infected with Ad-null; 18Q: infected with Ad-Htt-552-18Q; 100Q: infected with Ad-Htt-552-100Q (m.o.i. of 210). (C) Effects of expression of Htt-552 on cell viability following siRNA of LAMP-2A and Hsc70 (120 h). Following exposure of PC12 cells to virus vectors, cell viability was analyzed by the MTT assay at 120 h post-infection. Values were given as mean ± SD of 3 independent experiments. Null: infected with Ad-null; 18Q: infected with Ad-Htt-552-18Q; 100Q: infected with Ad-Htt-552-100Q (m.o.i. of 210). **P<0.01,*P<0.05 (compared with Htt without treatment). ##P<0.01, #P<0.05 (Htt-552-100Q compared with Htt-552-18Q). (D) Effects of expression of Htt-552 on cell cycle distribution following treatment with siRNA targeting LAMP-2A or Hsc70 (120 h). The cell cycle analysis and cell apoptosis were determined using FACS analysis as described in Methods and Materials. ,*P<0.05 (compared with Htt without treatment). ##P<0.01, #P<0.05 (Htt-552-100Q compared with Htt-552-18Q). (TIF) [file pone.0046834.s002.tif]

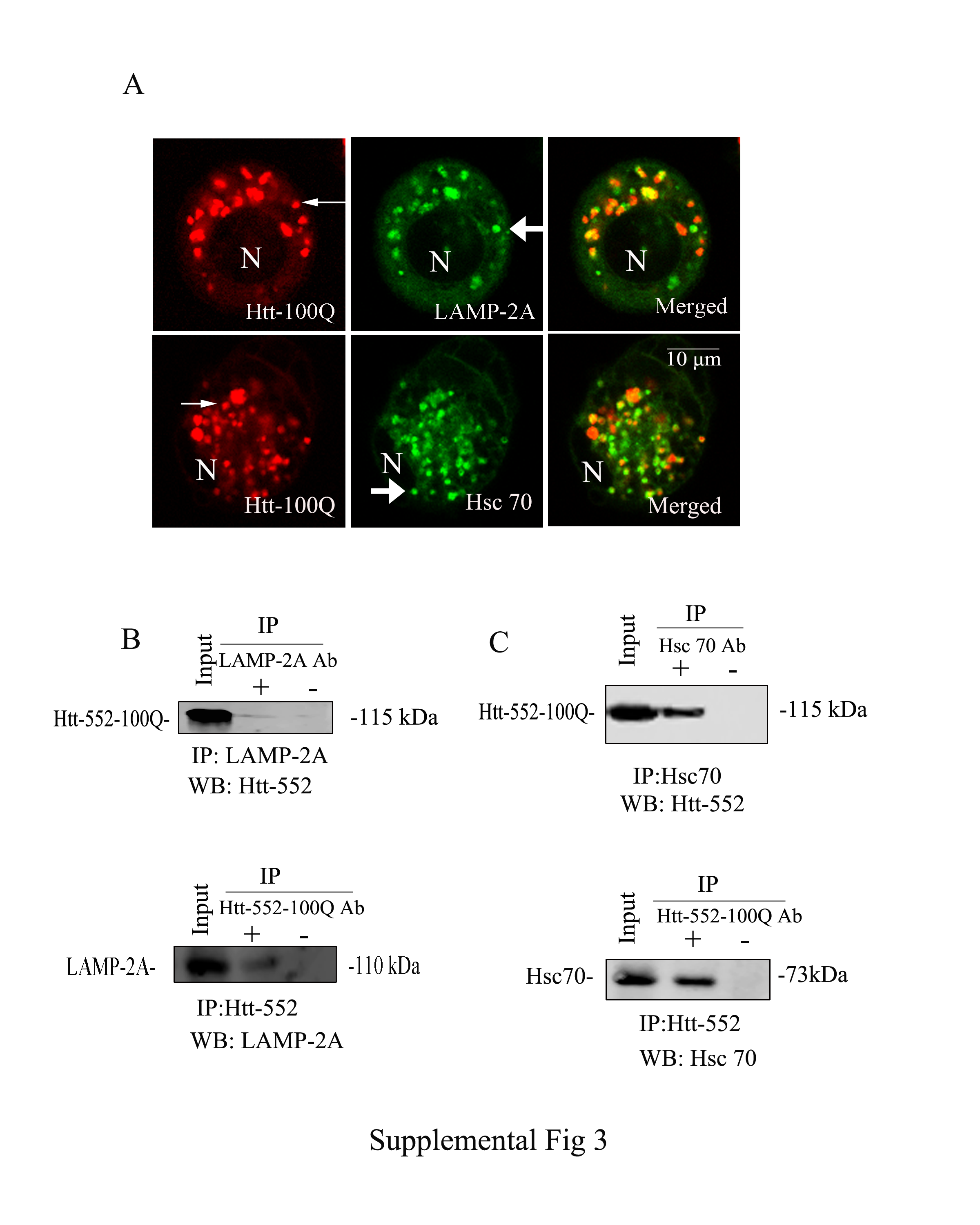

Supplement: Figure S3 — Association of Htt-552 and endogenous CMA component proteins. (A) Endogenous expressed LAMP-2A or Hsc70 co-localized with exogenous Htt-552-100Q in HeLa cells. Cells were transfected with Htt-552 for 48 h and fixed with methanol, blocked, and processed for double immunofluorescence with antibodies against Htt-552-100Q (red) Htt-552-100Q (red) and LAMP-2A (green) or Hsc70 (green). Merged images of both channels are shown at right. N: nucleus. Thin arrows point to Htt-552 immunoreactivity. Thick arrows point to lysosomes. The scale bar = 10 µm. (B) Co-immunoprecipitation of endogenous expressed LAMP-2A with exogenously expressed Htt-552-100Q. Upper panel: Cell lysates were subjected to immunoprecipitation with anti-Htt antibody or no primary antibody and Western blot analysis was performed with anti-LAMP-2A antibody. Lower panel: PC12 cell were transfected with Htt-552-100Q for 48 h. Cell lysates were subjected to immunoprecipitation with anti-LAMP-2A antibody or no primary antibody and Western blot analysis was performed with anti-Htt antibody. (C) Co-immunoprecipitation of endogenous expressed Hsc70 with exogenously expressed Htt-552-100Q. Upper panel: Cell lysates were subjected to immunoprecipitation with anti-Hsc70 antibody or no primary antibody and Western blot analysis was performed with anti-Htt antibody. Lower panels:PC12 cell were transfected with Htt-552-100Q for 48 h. Cell lysates were subjected to immunoprecipitation with anti-Htt antibody or no primary antibody and Western blot analysis was performed with anti-Hsc70 antibody. Ab: Antibody. (TIF) [file pone.0046834.s003.tif]
